# Pulmonary artery enlargement is associated with pulmonary hypertension and decreased survival in severe cystic fibrosis: A cohort study

**DOI:** 10.1371/journal.pone.0229173

**Published:** 2020-02-20

**Authors:** Aline N. Zouk, Swati Gulati, Dongqi Xing, Keith M. Wille, Steven M. Rowe, J. Michael Wells

**Affiliations:** 1 Division of Pulmonary, Allergy, and Critical Care Medicine, University of Alabama at Birmingham (UAB), Birmingham, AL, United States of America; 2 Gregory Fleming James Cystic Fibrosis Research Center, Birmingham, AL, United States of America; 3 UAB Lung Health Center, Birmingham, AL, United States of America; 4 Birmingham VA Medical Center, Birmingham, AL, United States of America; Medizinische Universitat Graz, AUSTRIA

## Abstract

**Background:**

Pulmonary artery (PA) enlargement, defined as pulmonary artery to ascending aorta diameter ratio (PA:A)>1 on computed tomography (CT), is a marker of pulmonary vascular disease in chronic lung diseases. PA enlargement is prevalent in cystic fibrosis (CF), but its relationship to hemodynamics and prognostic utility in severe CF are unknown. We hypothesized that the PA:A would have utility in identifying pulmonary hypertension (PH) in severe CF and that PA enlargement would be associated with reduced transplant-free survival.

**Methods:**

We conducted a retrospective study of adults with CF undergoing lung transplant evaluation at a single center between 2000 and 2015. CT, right heart catheterization (RHC), and clinical data were collected. The PA:A was measured from a single CT slice. We measured associations between PA:A and invasive hemodynamic parameters including PH defined as a mPAP ≥25mmHg using adjusted linear and logistic regression models. Kaplan-Meier and adjusted Cox regression models were used to measure associations between PA:A>1, RHC-defined PH, and transplant-free survival in severe CF.

**Results:**

We analyzed 78 adults with CF that had CT scans available for review, including 44 that also had RHC. RHC-defined PH defined as a mPAP ≥25mmHg was present in 36% of patients with CF undergoing transplant evaluation. The PA:A correlated with mPAP (r = 0.73; 95% CI 3.87–7.80; p<0.001) and PVR (r = 0.42, p = 0.005) and the PA:A>1 was an independent predictor of PH (aOR 4.50; 95% CI 1.05–19.2; p = 0.042). PA:A>1 was independently associated with increased hazards for death or transplant (aHR 2.69; 95% CI 1.41–5.14; P = 0.003). The presence of mPAP ≥25mmHg was independently associated with decreased survival in this cohort.

**Conclusions:**

PA enlargement is associated with pulmonary hemodynamics and PH in severe CF. PA enlargement is an independent prognostic indicator of PH and decreased survival in this population.

## Introduction

Cystic fibrosis (CF) is an autosomal recessive genetic disorder that leads to functional impairment in the cystic fibrosis transmembrane conductance regulator (CFTR) channel, leading to impaired mucociliary transport, recurrent infections, airflow obstruction, bronchiectasis, and altered gas exchange. Lung function impairment is strongly associated with morbidity and mortality [1, 2]. Other comorbid conditions including pulmonary hypertension (PH) further negatively impact survival in CF. Nearly 57% of individuals with CF undergoing lung transplant evaluation have already developed PH by the time they undergo testing [3]. In previous studies, the presence of PH was shown to be a strong independent predictor of morbidity and mortality in adults with CF, with the risk of mortality higher among patients with CF compared with other patient populations awaiting lung transplant [4, 5]. Even subclinical PH can have a negative impact on exercise capacity, perceived dyspnea, quality of life, and mortality [6, 7].

Right heart catheterization (RHC) is the gold standard for confirming PH; however, the technique’s invasiveness precludes its use on a regular basis in chronic lung diseases including CF. Thus, there is evolving interest in using non-invasive tools for assessing the presence of PH, such as computed tomography (CT), Doppler echocardiography and cardiac magnetic resonance imaging [8, 9]. CT-measured pulmonary artery enlargement defined by an elevated pulmonary artery diameter (PA) to ascending aorta diameter (A), PA:A, is a reliable imaging-based biomarker that detects PH [10–14], and can be obtained concomitant with cross-sectional pulmonary imaging that inform prognosis and diagnosis of complications. We previously reported that PA enlargement (PA:A>1) is highly prevalent in moderate-to-severe CF and individuals with PA enlargement were at a significantly higher risk for acute pulmonary exacerbation [15]. To our knowledge, there have been no studies exploring the association between PA:A and right-heart pressure via echocardiography or invasive hemodynamics from RHC. We hypothesized that the PA:A would have utility in identifying pulmonary hypertension (PH) in severe CF and that PA enlargement would be associated with reduced transplant-free survival.

## Materials and methods

### Ethics statement

The University of Alabama at Birmingham (UAB) Institutional Review Board (X150128006) approved this study. The IRB waived requirement for informed consent based on the nature of the investigation. None of the data were obtained from a vulnerable population (children, prisoners, or subjects with reduced mental capacity).

### Study design and population

Patients who underwent lung transplant evaluation at the University of Alabama at Birmingham (UAB) between January 1, 2000 and December 31, 2015 and carried a diagnosis of CF were identified using the UAB Lung Transplant Database. Subjects were enrolled if they had a diagnosis of CF with the presence of known CF-causing mutations and had measurable CT as part of lung transplantation work-up. Lung transplantation work-up was conducted on an outpatient basis in patients that were free from acute decompensation or exacerbations.

### Clinical characteristics

Demographic and clinical data were collected from the medical records at the time of transplant evaluation and included age, sex, CF genotype, forced expiratory volume at 1 second (FEV_1_), forced vital capacity (FVC), diffusion lung capacity of carbon monoxide (DLCO), body mass index (BMI), use of supplemental oxygen, extra-pulmonary CF manifestations, and prior infectious history from culture data. Data regarding whether lung transplantation occurred and the date of transplant were also collected. All patients who had lung transplant underwent bilateral lung transplantation. The date of death was recorded as retrieved through medical record review. Transplant-free survival was calculated from date of transplant evaluation.

### PA:A measurement

**Fig 1:**
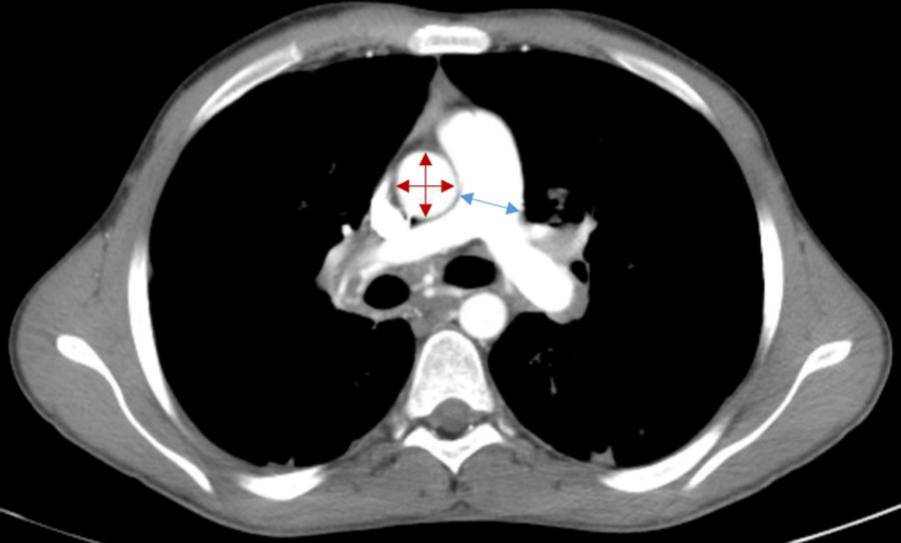
Pulmonary arterial enlargement in severe cystic fibrosis. A representative axial image of a CT scan at the level of the pulmonary artery (PA) bifurcation. The diameter of the PA is measured at the location indicated by the blue arrow. The diameter of the ascending aorta (A) is calculated using two perpendicular measurements indicated by the red arrows. PA enlargement (PA:A>1) is present in this image.

CT scans were reviewed separately by two independent blinded investigators (ANZ and SG) to assess for inter-observer agreement of PA:A measurements. The first investigator measured the PA, A, and PA:A from the same scan twice to assess for intra-observer reproducibility. The mean PA, A, and PA:A values were used for all analysis and calculations. PA:A was calculated by taking the ratio of the PA measured at the level of the PA bifurcation and the ascending aortic diameter measured from the same CT slice **(Fig 1)** using mediastinal windows with Philips iSite Enterprise Software (Koninklijke Philips N.V.) using previously published methods [10, 14, 15].

### Hemodynamic parameters

Hemodynamic variables were calculated or measured from the RHC, including mean right atrial pressure (RAP), mean pulmonary artery pressure (mPAP), cardiac output (CO) by thermodilution, cardiac index (CI), pulmonary vascular resistance (PVR), and pulmonary capillary wedge pressure (PW). PH was defined as a mPAP ≥25 mmHg on resting RHC.

### Statistical analysis

Continuous variables are presented as mean ± standard deviation and categorical variables as number and percentage. We considered missing data to be “missing at random” due to the retrospective nature of the study. Intra-class correlation coefficient (ICC) and Bland Altman analyses were used to measure the level of intra-observer and inter-observer agreement for measurement of PA, A, and PA:A. Pearson’s coefficient was used determine the correlation between PA:A and mPAP, as well as FEV1, FVC, DLCO and other clinical or hemodynamic variables. Unpaired t-test analysis was performed to compare mean differences between two groups for continuous variables and Chi-square or Fisher’s exact testing were used to compare differences for categorical variables, as appropriate. Linear regression analyses were used to measure the association between a z-score PA:A as a continuous variable mPAP, adjusting for age, sex, FEV1 percent predicted, and LTOT use, covariates previously shown to be associated with mPAP. Logistic regression models were used to determine associations between demographic characteristics, PA enlargement, and the presence of PH (mPAP ≥25mmHg). Covariates were selected based on known associations with CF disease severity. Kaplan-Meier survival curves were used to describe transplant free survival according to PA enlargement or PH as a function of time from the initial lung transplant evaluation. Kaplan-Meier curves were compared using the log-rank test. We then used Cox regression analysis to measure associations between PA enlargement, RHC-defined PH, and transplant free survival adjusting for age, sex, FEV1 percent predicted, and LTOT use. Patients who received lung transplantation were censored, as were those who died while awaiting transplant. Patient who were alive and had not received transplantation were censored at the end of data collection. A p-value <0.05 was considered statistically significant. All analyses were performed using IBM SPSS version 26.0 (SPSS Inc., Chicago, IL).

## Results

### Participant characteristics

**Fig 2:**
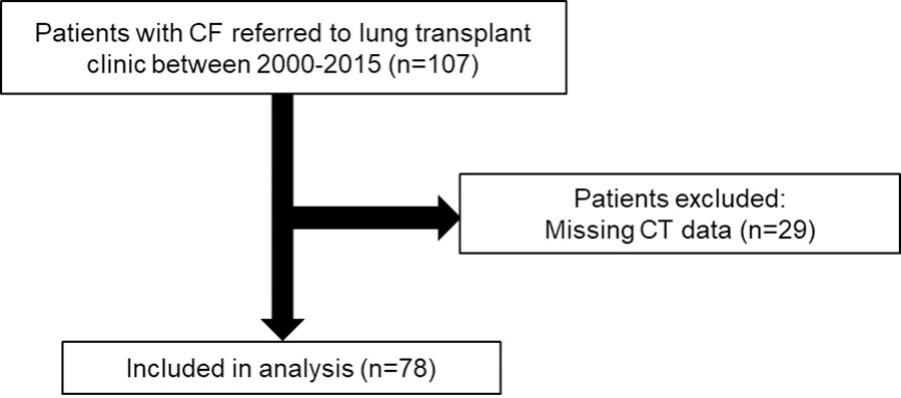
Study flow diagram. CF = cystic fibrosis. CT = contrast tomography. RHC = right heart catheterization.

There were 107 patients who carried a diagnosis of CF were referred for lung transplant evaluation between 2000 and 2015. Of those, 29 patients were excluded from analysis due to not having CT data, leaving a final cohort of 78 patients (**Fig 2**). From these, spirometric data were missing in n = 27; DLCO in n = 35; 6-minute walk distance in n = 41; and RHC data in n = 34. The cohort was 47% male, 97% White, and had a mean age of 28±10 years as shown in **Table 1**. All individuals in the cohort had two severe disease-causing mutations, including 37 (48%) Delta F508 homozygotes **(Table A in S1 File)**. Among individuals with culture data available for review, chronic *Pseudomonas* species was present in 96%, methicillin-resistant *Stapholococcus aureus* in 58%, and *Burkholderia cepacia* complex infection 14%. The mean FEV_1_ percent predicted was 24±6% and 67 (86%) patients used supplemental oxygen at the time of evaluation, confirming very severe underlying lung disease. In the 44 patients that had available RHC data, the average mPAP was 24±9 mmHg corresponding to 16 (36%) with PH defined by mPAP ≥25 mmHg. Of patients with RHC data, 13 (30%) had a mPAP 20–24 mmHg, 15 (34%) had mPAP 25–34 mmHg, and 3 (7%) had mPAP ≥35 mmHg suggesting the majority have mild-to-moderate PH.

**Table 1:** Baseline characteristics.

	Total (n = 78)	PA:A ≤1 (n = 41)	PA:A >1 (n = 37)	P-value
Age, years	28±10	29±10	27±9	0.41
Male sex	37 (47%)	24 (59%)	13 (35%)	0.04
White race	76 (97%)	41 (100%)	35 (95%)	0.13
BMI, kg/m^2^	18.5±3.6	17.9±4.2	19.1±2.8	0.24
LTOT use	67 (86%)	34 (83%)	33 (89%)	0.43
6MWD, m	349.7±93.7	364.3±88.2	336.0±99.2	0.37
*Pseudomonas* infection	48/50 (96%)	25/25 (100%)	23/25 (92%)	0.49
MRSA infection	29/50 (58%)	14/25 (56%)	15/25 (60%)	0.77
Burkholderia cepacia infection	7/50 (14%)	5/25 (20%)	2/25 (8%)	0.22
FEV_1_, L	0.84±0.27	0.86±0.25	0.82±0.29	0.65
FEV_1_, % predicted	24±6	25±5	24±7	0.65
FVC, % predicted	41±11	45±10	38±10	0.008
FEV_1_/FVC	0.48±0.12	0.45±0.10	0.51±0.13	0.11
DLCO, % predicted	53±12	56±12	49±11	0.07
PCWP, mmHg	9±4	8±5	10±3	0.10
mPAP, mmHg	24±9	20±6	30±10	<0.001
PVR, Wood Units	3.17±1.45	2.72±1.30	3.72±1.46	0.021
Cardiac Output, L/m	5.5±1.1	5.3±0.8	5.6±1.3	0.38

Data are expressed as mean±SD or n(%). Abbreviations: PA:A = pulmonary artery to ascending aorta diameter ratio; mPAP = mean pulmonary artery pressure; PCWP = pulmonary capillary wedge pressure; PVR = pulmonary vascular resistance; FEV1 = forced expiratory volume 1 sec; FVC = forced vital capacity; DLCO = diffusion capacity of lung carbon monoxide; LTOT = long term oxygen therapy; 6MWD = 6-minute walk distance; BMI = body mass index

We grouped patients based on the presence or absence of PA enlargement as shown in **Table 1**. Clinical characteristics including age, BMI, and 6MWD were similar between groups. There was a higher proportion of females compared to males in the PA enlargement group. Although FEV1 percent predicted and DLCO percent predicted were similar between groups, the FVC percent predicted was lower among CF patients with a PA:A>1 compared to those with a PA:A≤1. There were no differences in multi-drug resistant bacteria between PA:A groups.

### Reproducibility of PA:A measurement

In the 78 participants that had CT scan data available, the mean PA:A was 1.01±0.15, with 38 (48%) having PA enlargement (PA:A >1). The ICC for readings demonstrated excellent intra-observer agreement (R^2^ = 0.84, 95% CI 0.71–0.91, p<0.001) as well as inter-observer (R^2^ = 0.86, 95% CI 0.74–0.92, p<0.001). Bland Altman graphs representing inter and intra-observer variability are shown in **Fig A in S1 File**.

### The PA:A is associated with invasive hemodynamics and PH

As shown in **Table 1**, individuals in the PA:A>1 group had higher mPAP (30±10 mmHg vs 20±6 mmHg; P<0.001) and higher PVR (3.72±1.46 WU vs 2.72±1.30 WU; P = 0.021) compared to the PA:A ≤1 group. Otherwise, hemodynamic parameters including PW or CO were not statistically different between groups. There were significant correlations between the PA:A and mPAP (r = 0.73, p<0.001), PVR (r = 0.42, p = 0.005), but not PW (r = 0.26, p = 0.09). There were no statistically significant correlations between PA:A and FEV1, FVC, or DLCO. The PA:A was not associated with other clinical or hemodynamic variables including 6MWD or BMI. In linear regression models adjusted for age, sex, FEV_1_, and LTOT use, each standard deviation increase in PA:A was associated with a 6 (Standard Error 1) mmHg increase in mPAP (P<0.001).

**Fig 3:**
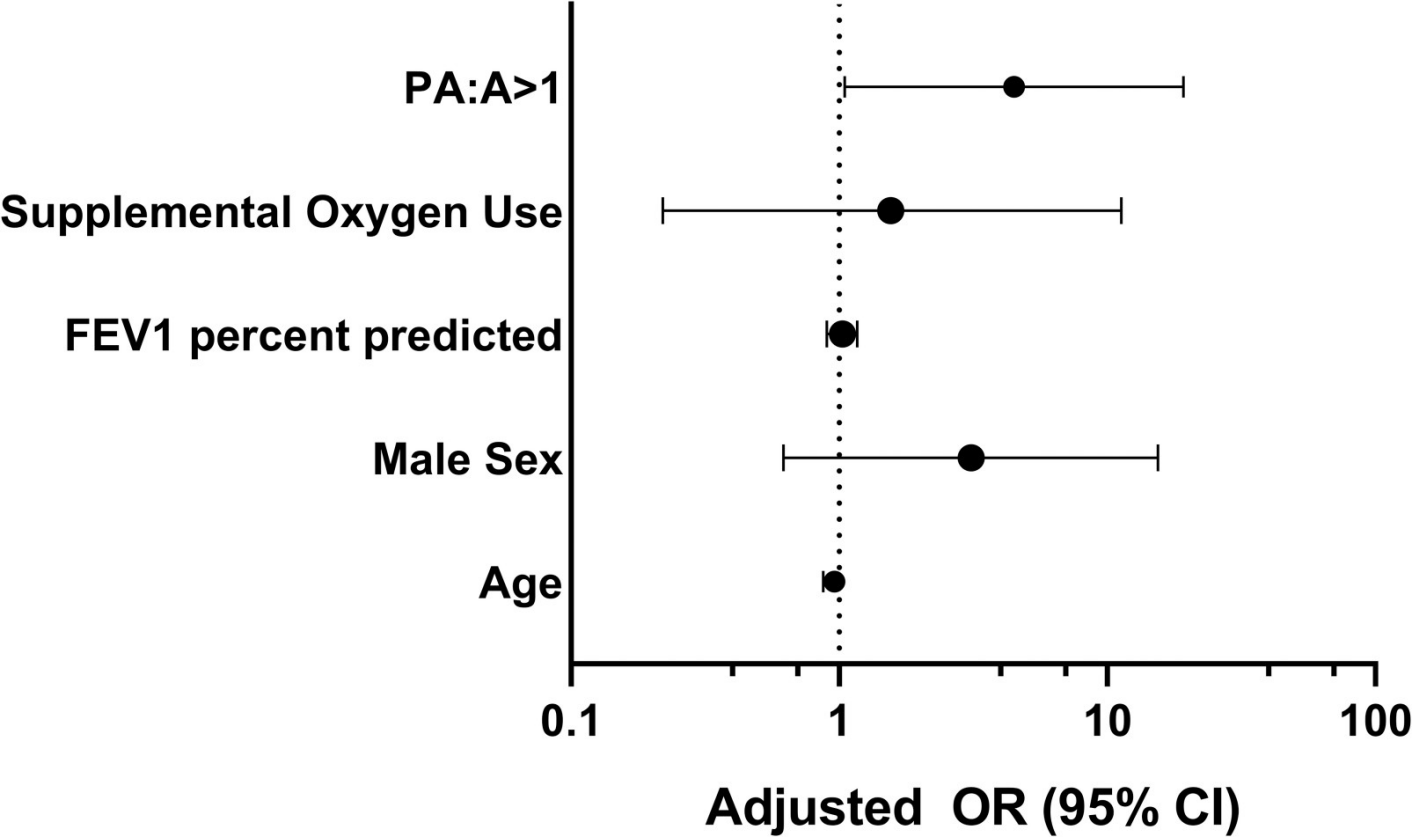
Associations between PA enlargement and pulmonary hypertension measured at right-heart catheterization in cystic fibrosis. PA enlargement was associated with PH defined by mPAP ≥25 mmHg (*P<0.05) in logistic regression models adjusted for age, sex, FEV1 percent predicted, and supplemental oxygen use. Abbreviations: CI = confidence interval; FEV1 = forced expiratory volume in 1-second; PA:A = pulmonary artery diameter to aorta diameter ratio.

We then tested the utility of the PA:A in predicting PH defined as mPAP ≥25mmHg. PA enlargement was associated with PH in Fisher’s exact testing (p = 0.029). In logistic regression models adjusting for age, sex, FEV1 percent predicted, and LTOT use, the PA:A was associated with PH (aOR 4.50; 95% CI 1.05–19.2; p = 0.042) as shown in **Fig 3**.

### PA enlargement and RHC-defined PH are associated with decreased transplant-free survival

**Fig 4:**
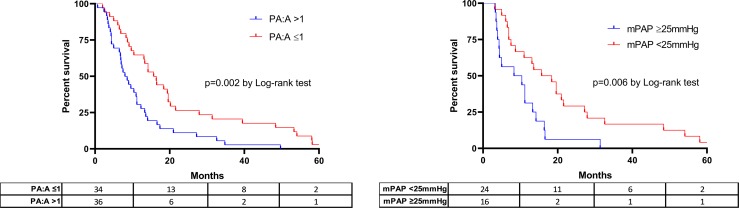
Kaplan-Meier curves for transplant-free survival in severe cystic fibrosis. Survival according to the presence or absence of A) PA:A>1; or B) mPAP ≥25mmHg. The numbers of subjects at risk are displayed below each figure panel. Abbreviations: mPAP = mean pulmonary artery pressure; PA:A = pulmonary artery to ascending aorta diameter ratio; PH = pulmonary hypertension.

In Kaplan-Meier analyses, patients with severe CF and PA enlargement had a 7-month shorter median survival time compared to those without PA enlargement (8.0 [95%CI 5.2–10.7] versus 15.7 [95% CI 12.3–19.0] months, P = 0.002 by log-rank) as shown in **Fig 4A**. PA enlargement remained a significant independent predictor of death or lung transplant (HR 2.69; 95% CI 1.41–5.14; P = 0.003) in a Cox proportional hazard model adjusted for age, sex, FEV1 and LTOT (**Table B in S1 File**). In a separate Kaplan-Meier survival model shown in **Fig 4B**, the presence of PH defined as a mPAP ≥25 mmHg was associated with shorter median transplant-free survival (8.3 [95%CI 0.00–18.9] versus 15.7 [95%CI 7.9–23.4] months; P = 0.006 by log-rank testing) compared to CF patients without PH.

## Discussion

Among patients with severe CF undergoing lung transplant evaluation, the PA:A correlated with invasive hemodynamic variables obtained by RHC and PA enlargement was associated with the presence of PH as well as transplant-free survival. To our knowledge, this is the first study to examine the relationship between PA:A and invasive hemodynamics in this population of severe CF. These findings provide the basis for a novel, reproducible, and easily obtainable measurement for suggesting PH in CF, where other non-invasive tools such as echocardiography have been shown to be sub-optimal in assessing pulmonary arterial pressures in this subgroup of patients [16].

In this cohort, the inter-observer and intra-observer reproducibility for measuring PA:A was excellent, consistent with findings in other lung diseases [10, 12, 14, 15, 17, 18]. The mPAP was higher in subjects with a PA enlargement compared to those with a PA:A≤1. We did not find a correlation between PA:A and FEV1, DLCO, or supplemental oxygen use, though we observed a modest correlation with FVC. These findings suggest that while lung parenchymal destruction and hypoxia-mediated PH contribute significantly to PA enlargement, other mechanisms such as pulmonary vascular remodeling, parenchymal changes of the lung, and cardiac dysfunction may also be playing a role [10, 19, 20]. This may also reflect the fact that FEV_1_ becomes a less dynamic marker in end-stage CF as compared to FVC, which remains responsive in severe obstruction.

Current recommendations for the evaluation of patients with suspected PH begin with an echocardiogram as an initial screening test to estimate the right ventricular systolic pressure and to evaluate for right heart chamber enlargement [21]. While multiple studies have shown a good correlation between echocardiographic and RHC measurements [22–24], it has its limitations in patients with obstructive lung diseases [16]. Hyperinflation from severe obstructive lung disease causes increase in intrathoracic gas and thoracic cage diameter, which in turn increases the distance between the probe and heart. This causes difficulty in detecting and obtaining a clear signal of regurgitant flow, poor acoustic windows, and poor visualization of the right heart [16, 24]. In one study by Arcasoy et al. on patients with advanced lung disease, Doppler echocardiography estimated PASP in only 44% of the cohort, did not correlate with RHC measurements, and had low sensitivity of 76% [16].

The presence of PH in CF is associated with increased morbidity and mortality [4], and worse clinical outcomes including shorter 6-minute walk distance, increased LTOT use, and worse pulmonary function [25], all of which lead to decreased quality of life and increased health care cost. Even subclinical PH leads to more pronounced exercise impairment and dyspnea [6]. However, the prevalence of PH in CF is largely unknown. It is estimated to be between 25–60% [3, 5, 25–28]. PH defined by mPAP ≥25 mmHg was present in 36% of our cohort that underwent RHC, a finding that is comparable to the prevalence of 38% reported by other investigators [5, 26]. In our cohort, PA enlargement (PA:A >1) was present in 48% of individuals undergoing lung transplant evaluation, observations similar to what we have observed in a less-severe CF population [15].

We found that each standard deviation increase in PA:A corresponded to a 6±1 mmHg increase in mPAP and the presence of PA enlargement was associated with PH. These findings correspond to similar observations in patients with chronic obstructive lung disease (COPD) [10, 12] and idiopathic pulmonary fibrosis (IPF) [17, 18]. A PA:A>1 was indeed a strong independent predictor of PH, suggesting its strong role as non-invasive screening test for PH in patients with severe CF.

While chronic hypoxia likely accounts for most of the vascular remodeling and altered hemodynamics, other factors could play a role in CF-relate PH pathogenesis. These include chronic inflammation and CFTR dysfunction. Giacchi and colleagues previously demonstrated correlations between PA pressures measured by echocardiography and erythrocyte sedimentation rate and c-reactive protein, suggesting that chronic inflammation may precede pulmonary vascular disease [29]. CFTR could also be directly involved in pulmonary vascular remodeling. The role of CFTR in the regulation of the vascular tone of smooth muscle cells directly through channel activation has been described in the literature [30]. CFTR in intrapulmonary arteries leads to endothelium-independent vasorelaxation through other indirect pathways such as nitric oxide generation and alteration of lipid handling [31, 32]. While we previously observed that increased sweat chloride concentrations, a surrogate marker of CFTR function, was indeed associated with PA:A, further supporting this hypothesis [15], we did not have sufficient sweat chloride measurements or other tests of CFTR dysfunction in this cohort to test this hypothesis. Future studies are needed to determine the exact role CFTR dysfunction has on pulmonary vascular constriction in populations of CF with less severe disease, as this could prove to be a potential therapeutic target before irreversible arterial remodeling occurs.

Efforts to define clinical characteristics associated with survival in patients with CF have been unsuccessful, with no single factor independently predictive of poor outcomes [2, 33]. Although there is not definitive evidence, FEV1 <30%, rapid decline in lung function, and increased number of exacerbations and hospitalizations are all considered concerning factors that play a role in physicians’ decision for timing of transplant evaluation referral [34]. Despite being an internationally recommended indication for lung transplant evaluation, the role of PH as a trigger for referral remains underutilized. In one study in 2015, only 54% of medical directors of Cystic Fibrosis Foundation accredited-care centers in the United States considered PH a trigger for lung transplant evaluation referral [34]. The association of PH and poor outcomes in CF remains controversial. Whereas studies in the past did not show association between PH and survival [25–27], we found that patients with mPAP ≥25 mmHg have a higher risk of mortality and poorer outcomes, confirming the findings reported by Hayes et al [28]. Similarly, we found that there was an association between PA enlargement defined and transplant-free survival, and that the presence of PA enlargement portends a worse prognosis. We therefore report that the PA:A provides valuable information to providers evaluating patients for lung transplantation by providing a screening test to identify patients at highest risk for having underlying PH that may benefit from undergoing RHC, improving the allocation process for donor lungs. Moreover, its presence is associated with increased mortality and perhaps argues for a more urgent need for lung transplantation in this population.

The main limitations of our study are its retrospective nature, single center design, limited number of RHC for the cohort, and small sample size. Despite the small sample size, however, the relationships between PA:A and mPAP remained significant when controlling for other variables, emphasizing the strong role an enlarged PA:A plays in predicting PH. Another limitation is that our cohort was comprised of patients with severe obstructive lung disease as evidence by low FEV1 and high LTOT use, and we cannot therefore generalize our results to adults with mild or moderate CF. However, we have previously shown that PA enlargement is highly prevalent in CF with less severe lung disease [15]. PA:A may still have a role in identifying PH in a less severely ill population and this should be explored in future studies. Additionally, we do not have data on acute exacerbations in this cohort. We have previously demonstrated relationships between PA enlargement and exacerbations of CF and this is worth further exploration in a larger study size to further elucidate how exacerbations may play a role in CF vasculopathy, particularly among individuals with very severe disease. A further limitation is that the current study was focused entirely on changes to the central vasculature on CT. It is possible that other findings on CT imaging, including markers of RV function or strain (including the RV/LV ratio) could also have meaningful associations with hemodynamics and outcomes in this population.

## Conclusions

We demonstrated that PA:A is a widely-available, reproducible, non-invasive tool that can be used as an independent predictor of invasive hemodynamics and PH in CF patients undergoing lung transplant evaluation. The presence of PA enlargement is associated with reduced survival in this population. These findings suggest a potential role for PA:A as a tool for detection of PH and timely referral for transplant evaluation. These findings should be confirmed in future, multi-center studies to further assess the utility of PA enlargement as a screening tool for PH in CF.

## Supporting information

S1 File **Fig A.** Bland-Altman plot for PA:A reproducibility. Bland-Altman plots for intra-observer variability using two measurements from the same reader (Left Panel) and inter-observer variability using measurements from two independent readers (Right Panel).
**Table A.** Gene mutation frequency in the CF cohort undergoing lung transplant evaluation.
**Table B.** Multivariable Cox Proportional Hazards Model Results.
(DOCX)

## Abbreviations

PA: pulmonary artery
CT: computed tomography
CF: cystic fibrosis
PH: pulmonary hypertension
PA:A: pulmonary artery to aorta ratio
mPAP: mean pulmonary artery pressure

## References

[1] Kerem E, Reisman J, Corey M, Canny GJ, Levison H. Prediction of mortality in patients with cystic fibrosis. N Engl J Med. 1992;326(18):1187–91. 10.1056/NEJM199204303261804 1285737

[2] Belkin RA, Henig NR, Singer LG, Chaparro C, Rubenstein RC, Xie SX, et al Risk factors for death of patients with cystic fibrosis awaiting lung transplantation. Am J Respir Crit Care Med. 2006;173(6):659–66. 10.1164/rccm.200410-1369OC 16387803 PMC2662949

[3] Hayes D Jr., Higgins RS, Kirkby S, McCoy KS, Wehr AM, Lehman AM, et al Impact of pulmonary hypertension on survival in patients with cystic fibrosis undergoing lung transplantation: an analysis of the UNOS registry. J Cyst Fibros. 2014;13(4):416–23. 10.1016/j.jcf.2013.12.004 24388063

[4] Hayes D Jr., Tobias JD, Mansour HM, Kirkby S, McCoy KS, Daniels CJ, et al Pulmonary hypertension in cystic fibrosis with advanced lung disease. Am J Respir Crit Care Med. 2014;190(8):898–905. 10.1164/rccm.201407-1382OC 25222938

[5] Venuta F, Tonelli AR, Anile M, Diso D, De Giacomo T, Ruberto F, et al Pulmonary hypertension is associated with higher mortality in cystic fibrosis patients awaiting lung transplantation. J Cardiovasc Surg (Torino). 2012;53(6):817–20. 23207567

[6] Manika K, Pitsiou GG, Boutou AK, Tsaoussis V, Chavouzis N, Antoniou M, et al The Impact of Pulmonary Arterial Pressure on Exercise Capacity in Mild-to-Moderate Cystic Fibrosis: A Case Control Study. Pulm Med. 2012;2012:252345 10.1155/2012/252345 22900167 PMC3414060

[7] Fraser KL, Tullis DE, Sasson Z, Hyland RH, Thornley KS, Hanly PJ. Pulmonary hypertension and cardiac function in adult cystic fibrosis: role of hypoxemia. Chest. 1999;115(5):1321–8. 10.1378/chest.115.5.1321 10334147

[8] Benza R, Biederman R, Murali S, Gupta H. Role of cardiac magnetic resonance imaging in the management of patients with pulmonary arterial hypertension. J Am Coll Cardiol. 2008;52(21):1683–92. 10.1016/j.jacc.2008.08.033 19007687

[9] Kovacs G, Reiter G, Reiter U, Rienmuller R, Peacock A, Olschewski H. The emerging role of magnetic resonance imaging in the diagnosis and management of pulmonary hypertension. Respiration. 2008;76(4):458–70. 10.1159/000158548 19018164

[10] Iyer AS, Wells JM, Vishin S, Bhatt SP, Wille KM, Dransfield MT. CT scan-measured pulmonary artery to aorta ratio and echocardiography for detecting pulmonary hypertension in severe COPD. Chest. 2014;145(4):824–32. 10.1378/chest.13-1422 24114440 PMC3971971

[11] Karakus G, Kammerlander AA, Aschauer S, Marzluf BA, Zotter-Tufaro C, Bachmann A, et al Pulmonary artery to aorta ratio for the detection of pulmonary hypertension: cardiovascular magnetic resonance and invasive hemodynamics in heart failure with preserved ejection fraction. J Cardiovasc Magn Reson. 2015;17:79 10.1186/s12968-015-0184-3 26318496 PMC4553215

[12] Shin S, King CS, Brown AW, Albano MC, Atkins M, Sheridan MJ, et al Pulmonary artery size as a predictor of pulmonary hypertension and outcomes in patients with chronic obstructive pulmonary disease. Respir Med. 2014;108(11):1626–32. 10.1016/j.rmed.2014.08.009 25225149

[13] Wells JM, Morrison JB, Bhatt SP, Nath H, Dransfield MT. Pulmonary Artery Enlargement Is Associated With Cardiac Injury During Severe Exacerbations of COPD. Chest. 2016;149(5):1197–204. 10.1378/chest.15-1504 26501747 PMC4944777

[14] Wells JM, Washko GR, Han MK, Abbas N, Nath H, Mamary AJ, et al Pulmonary arterial enlargement and acute exacerbations of COPD. N Engl J Med. 2012;367(10):913–21. 10.1056/NEJMoa1203830 22938715 PMC3690810

[15] Wells JM, Farris RF, Gosdin TA, Dransfield MT, Wood ME, Bell SC, et al Pulmonary artery enlargement and cystic fibrosis pulmonary exacerbations: a cohort study. Lancet Respir Med. 2016;4(8):636–45. 10.1016/S2213-2600(16)30105-9 27298019 PMC5672808

[16] Arcasoy SM, Christie JD, Ferrari VA, Sutton MS, Zisman DA, Blumenthal NP, et al Echocardiographic assessment of pulmonary hypertension in patients with advanced lung disease. Am J Respir Crit Care Med. 2003;167(5):735–40. 10.1164/rccm.200210-1130OC 12480614

[17] Yagi M, Taniguchi H, Kondoh Y, Ando M, Kimura T, Kataoka K, et al CT-determined pulmonary artery to aorta ratio as a predictor of elevated pulmonary artery pressure and survival in idiopathic pulmonary fibrosis. Respirology. 2017;22(7):1393–9. 10.1111/resp.13066 28488784

[18] Shin S, King CS, Puri N, Shlobin OA, Brown AW, Ahmad S, et al Pulmonary artery size as a predictor of outcomes in idiopathic pulmonary fibrosis. Eur Respir J. 2016;47(5):1445–51. 10.1183/13993003.01532-2015 26846836

[19] Estepar RS, Kinney GL, Black-Shinn JL, Bowler RP, Kindlmann GL, Ross JC, et al Computed tomographic measures of pulmonary vascular morphology in smokers and their clinical implications. Am J Respir Crit Care Med. 2013;188(2):231–9. 10.1164/rccm.201301-0162OC 23656466 PMC3778757

[20] Nakanishi R, Rana JS, Shalev A, Gransar H, Hayes SW, Labounty TM, et al Mortality risk as a function of the ratio of pulmonary trunk to ascending aorta diameter in patients with suspected coronary artery disease. Am J Cardiol. 2013;111(9):1259–63. 10.1016/j.amjcard.2013.01.266 23415638

[21] Galie N, Hoeper MM, Humbert M, Torbicki A, Vachiery JL, Barbera JA, et al Guidelines for the diagnosis and treatment of pulmonary hypertension: the Task Force for the Diagnosis and Treatment of Pulmonary Hypertension of the European Society of Cardiology (ESC) and the European Respiratory Society (ERS), endorsed by the International Society of Heart and Lung Transplantation (ISHLT). Eur Heart J. 2009;30(20):2493–537. 10.1093/eurheartj/ehp297 19713419

[22] Janda S, Shahidi N, Gin K, Swiston J. Diagnostic accuracy of echocardiography for pulmonary hypertension: a systematic review and meta-analysis. Heart. 2011;97(8):612–22. 10.1136/hrt.2010.212084 21357375

[23] D'Alto M, Romeo E, Argiento P, D'Andrea A, Vanderpool R, Correra A, et al Accuracy and precision of echocardiography versus right heart catheterization for the assessment of pulmonary hypertension. Int J Cardiol. 2013;168(4):4058–62. 10.1016/j.ijcard.2013.07.005 23890907

[24] Fisher MR, Criner GJ, Fishman AP, Hassoun PM, Minai OA, Scharf SM, et al Estimating pulmonary artery pressures by echocardiography in patients with emphysema. Eur Respir J. 2007;30(5):914–21. 10.1183/09031936.00033007 17652313

[25] Belle-van Meerkerk G, Cramer MJ, Kwakkel-van Erp JM, Nugroho MA, Tahri S, de Valk HW, et al Pulmonary hypertension is a mild comorbidity in end-stage cystic fibrosis patients. J Heart Lung Transplant. 2013;32(6):609–14. 10.1016/j.healun.2013.03.006 23582476

[26] Tonelli AR, Fernandez-Bussy S, Lodhi S, Akindipe OA, Carrie RD, Hamilton K, et al Prevalence of pulmonary hypertension in end-stage cystic fibrosis and correlation with survival. J Heart Lung Transplant. 2010;29(8):865–72. 10.1016/j.healun.2010.04.006 20466565

[27] Scarsini R, Prioli MA, Milano EG, Castellani C, Pesarini G, Assael BM, et al Hemodynamic predictors of long term survival in end stage cystic fibrosis. Int J Cardiol. 2016;202:221–5. 10.1016/j.ijcard.2015.09.009 26397415

[28] Hayes D Jr., Tumin D, Daniels CJ, McCoy KS, Mansour HM, Tobias JD, et al Pulmonary Artery Pressure and Benefit of Lung Transplantation in Adult Cystic Fibrosis Patients. Ann Thorac Surg. 2016;101(3):1104–9. 10.1016/j.athoracsur.2015.09.086 26687141

[29] Giacchi V, Rotolo N, Amato B, Di Dio G, Betta P, La Rosa M, et al Heart involvement in children and adults with cystic fibrosis: correlation with pulmonary indexes and inflammation markers. Heart Lung Circ. 2015;24(10):1002–10. 10.1016/j.hlc.2015.03.006 25911142

[30] Robert R, Thoreau V, Norez C, Cantereau A, Kitzis A, Mettey Y, et al Regulation of the cystic fibrosis transmembrane conductance regulator channel by beta-adrenergic agonists and vasoactive intestinal peptide in rat smooth muscle cells and its role in vasorelaxation. J Biol Chem. 2004;279(20):21160–8. 10.1074/jbc.M312199200 15020588

[31] Robert R, Savineau JP, Norez C, Becq F, Guibert C. Expression and function of cystic fibrosis transmembrane conductance regulator in rat intrapulmonary arteries. Eur Respir J. 2007;30(5):857–64. 10.1183/09031936.00060007 17596272

[32] Tabeling C, Yu H, Wang L, Ranke H, Goldenberg NM, Zabini D, et al CFTR and sphingolipids mediate hypoxic pulmonary vasoconstriction. Proc Natl Acad Sci U S A. 2015;112(13):E1614–23. 10.1073/pnas.1421190112 25829545 PMC4386337

[33] Mayer-Hamblett N, Rosenfeld M, Emerson J, Goss CH, Aitken ML. Developing cystic fibrosis lung transplant referral criteria using predictors of 2-year mortality. Am J Respir Crit Care Med. 2002;166(12 Pt 1):1550–5. 12406843 10.1164/rccm.200202-087OC

[34] Ramos KJ, Somayaji R, Lease ED, Goss CH, Aitken ML. Cystic fibrosis physicians' perspectives on the timing of referral for lung transplant evaluation: a survey of physicians in the United States. BMC Pulm Med. 2017;17(1):21 10.1186/s12890-017-0367-9 28103851 PMC5248524

