# Supplementary material for: Pulmonary artery enlargement is associated with pulmonary hypertension and decreased survival in severe cystic fibrosis: A cohort study
Source: PLoS One. 2020 Feb 20;15(2):e0229173. doi: 10.1371/journal.pone.0229173 (PMC7032721; doi:10.1371/journal.pone.0229173)
Supplement: S1 File — Fig A. Bland-Altman plot for PA:A reproducibility. Bland-Altman plots for intra-observer variability using two measurements from the same reader (Left Panel) and inter-observer variability using measurements from two independent readers (Right Panel). Table A. Gene mutation frequency in the CF cohort undergoing lung transplant evaluation. Table B. Multivariable Cox Proportional Hazards Model Results. (DOCX) [file pone.0229173.s001.docx]

**Supporting Information File**

Pulmonary Artery Enlargement is Associated with Pulmonary Hypertension and Decreased Survival in Severe Cystic Fibrosis: A Cohort Study

Aline N. Zouk, MD, et. al.


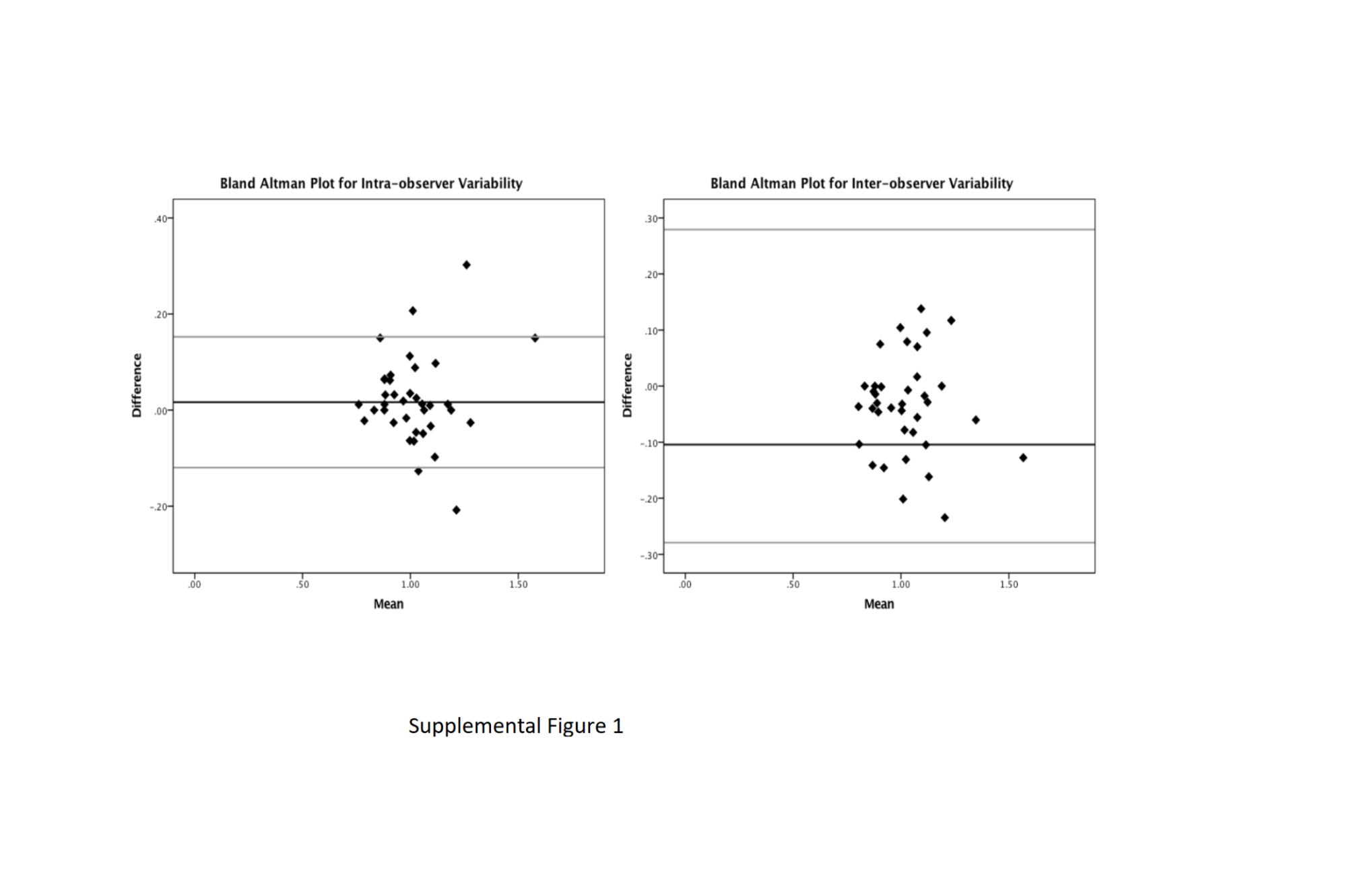
**Fig A. Bland-Altman plot for PA:A reproducibility.**

Bland-Altman plots for intra-observer variability using two measurements from the same reader (Left Panel) and inter-observer variability using measurements from two independent readers (Right Panel).

**Table A.** Gene mutation frequency in the CF cohort undergoing lung transplant evaluation

| **Genotype** | **Percent of cohort with mutation** |
| --- | --- |
| **DeltaF508** | 48% |
| **p.R1162X** | 1.7% |
| **p.G551D** | 1.7% |
| **p.G542X** | 3.7% |
| **p.Q493X** | 1.7% |
| **R117H** | 1.7% |
| **c.3849+10kbC>T** | 3.7% |
| **c.2052_2053insA** | 1.7% |
| **Unknown** | 71% |

**Table B**. Multivariable Cox Proportional Hazards Model Results

|  | **Hazards Ratio** | **95% CI** | **p-value** |
| --- | --- | --- | --- |
| PA:A >1 | 2.69 | 1.41-5.14 | 0.003 |
| Age | 1.01 | 0.98-1.05 | 0.51 |
| Male sex | 0.97 | 0.52-1.83 | 0.93 |
| FEV1 percent predicted | 0.99 | 0.94-1.07 | 0.96 |
| Supplemental oxygen use | 1.14 | 0.50-2.62 | 0.75 |

Definitions: PA:A = pulmonary artery diameter to aorta diameter ratio; FEV1 = forced expiratory volume in 1-second
